# Biochemical significance of limonene and its metabolites: future prospects for designing and developing highly potent anticancer drugs

**DOI:** 10.1042/BSR20181253

**Published:** 2018-11-14

**Authors:** Yusif M. Mukhtar, Michael Adu-Frimpong, Ximing Xu, Jiangnan Yu

**Affiliations:** 1 Department of Pharmaceutics and Tissue Engineering, School of pharmacy, Jiangsu University, 301 Xuefu Road, Zhenjiang 212013, Jiangsu Province, P.R. China; 2 Department of Basic and Biomedical Sciences, College of Health and Well-Being, P. O. Box 9, Kintampo, Ghana

**Keywords:** limonene, metabolites, monoterpenes, pharmacological

## Abstract

Monocyclic monoterpenes have been recognized as useful pharmacological ingredients due to their ability to treat numerous diseases. Limonene and perillyl alcohol as well as their metabolites (especially perillic acid and its methyl ester) possess bioactivities such as antitumor, antiviral, anti-inflammatory, and antibacterial agents. These therapeutic properties have been well documented. Based on the aforementioned biological properties of limonene and its metabolites, their structural modification and development into effective drugs could be rewarding. However, utilization of these monocyclic monoterpenes as scaffolds for the design and developments of more effective chemoprotective agents has not received the needed attention by medicinal scientists. Recently, some derivatives of limonene metabolites have been synthesized. Nonetheless, there have been no thorough studies on their pharmacokinetic and pharmacodynamic properties as well as their inhibition against isoprenylation enzymes. In this review, recent research progress in the biochemical significance of limonene and its metabolites was summarized with emphasis on their antitumor effects. Future prospects of these bioactive monoterpenes for drug design and development are also highlighted.

## Introduction

**Figure 1:**
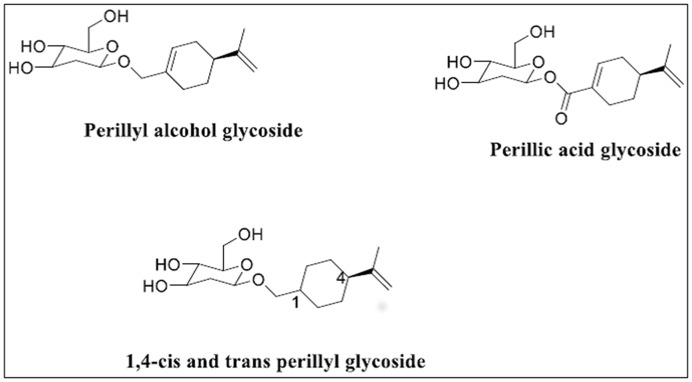
Naturally occurring perillyl glycosides

In recent decades natural products continue to attract intense attention due to their various bioactivities. Nowadays, most of the drugs on the market are inspired by or derived from natural sources [1]. As one of the most common terpenes in nature, limonene is a major component of essential natural products such as citrus rind oil, dill oil, cumin oil, neroli, bergamot, and caraway. On the other hand, perillyl alcohol (POH) is a metabolite of limonene as well as a naturally occurring monoterpene found in essential oils of mints, cherries, lavenders, lemongrass, sage, cranberries, perilla, wild bergamot, ginger grass, savin, caraway, and celery seeds [2,3]. Perillic acid (PA) is one of the key metabolites of limonene and POH in the human plasma [4]. Naturally, PA exists in the glycoside form (Figure 1) [5] but can also be produced via bioconversion of limonene and perillyl alcohol [6,7].

Limonene and its metabolites have demonstrated numerous biochemical effects as chemotherapeutic agents. They are recognized as anticancer agents owing to their ability to induce apoptosis by up-regulating of pro-apoptotic factors and down-regulating anti-apoptotic factors [13,17]. They have also been involved in a range of functions including inhibition of small G protein isoprenylation plus induction of proto-oncogenes [8], inhibition of Na^+^/K^+^ ATPase [9], disruption of the telomerase catalytic subunit reverse transcriptase (hTERT)-mechanistic target of rapamycin (mTOR)-regulatory associated protein of mTOR (RAPTOR) [(hTERT−mTOR−RAPTOR)] protein complex [10], suppression of 3-hydroxy-3-methylglutaryl of 4E-BP1(Ser65) phosphorylation [11], and cap-dependent translation [12].

**Figure 2:**
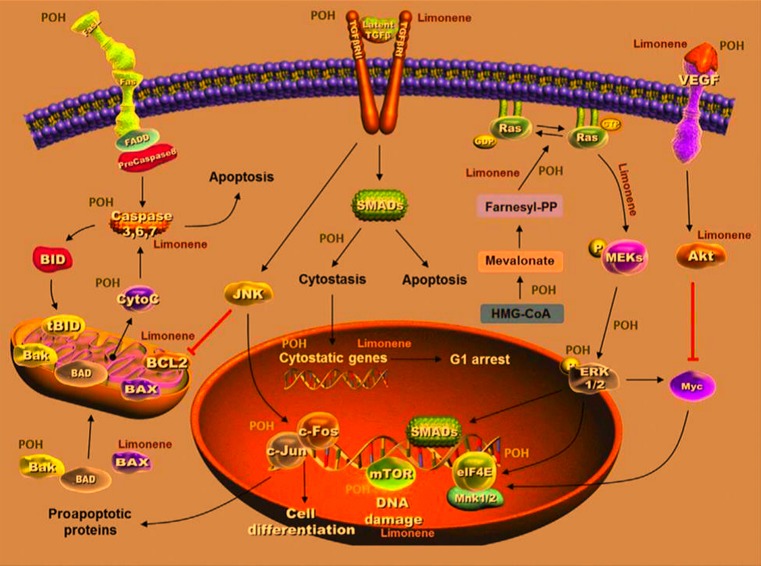
Inhibitory and stimulatory roles of limonene and POH in some key pathways involved in tumor growth and death [19]

Limonene and POH play various inhibitory and stimulatory roles in some key pathways involved in tumor progression and regression. These natural bioactive compounds also play an important role in regulation of cell death. Limonene exerted its effects by up-regulation of B-cell lymphoma-2 (Bcl-2)-associated X protein (BAX), cytochrome *c* release, cysteine-aspartic proteases (caspase)-3, caspase-9, transforming growth factor β (TGF-β), and down-regulation of anti-apoptotic Bcl-2 [13]. On the other hand, POH also up-regulates Bcl-2 homologous antagonist/killer (Bak), caspase-3, FasL, TGF-β, c-fos, and c-Jun as well as blocks extracellular signal-regulated kinase (ERK)-1/2 phosphorylation alongside mitogen/extracellular signal-regulated kinase (Mek)–extracellular signal-regulated kinase (Erk) pathway [14,15]. Moreover, both limonene and POH could inhibit tumor progression through down-regulation of basal production of vascular endothelial growth factor (VEGF) in cancer cells [16]. Furthermore, they also suppressed mevalonate pathway as well as isoprenylation of small G proteins, leading to tumor regression [17,18] (Figure 2).

**Figure 3:**
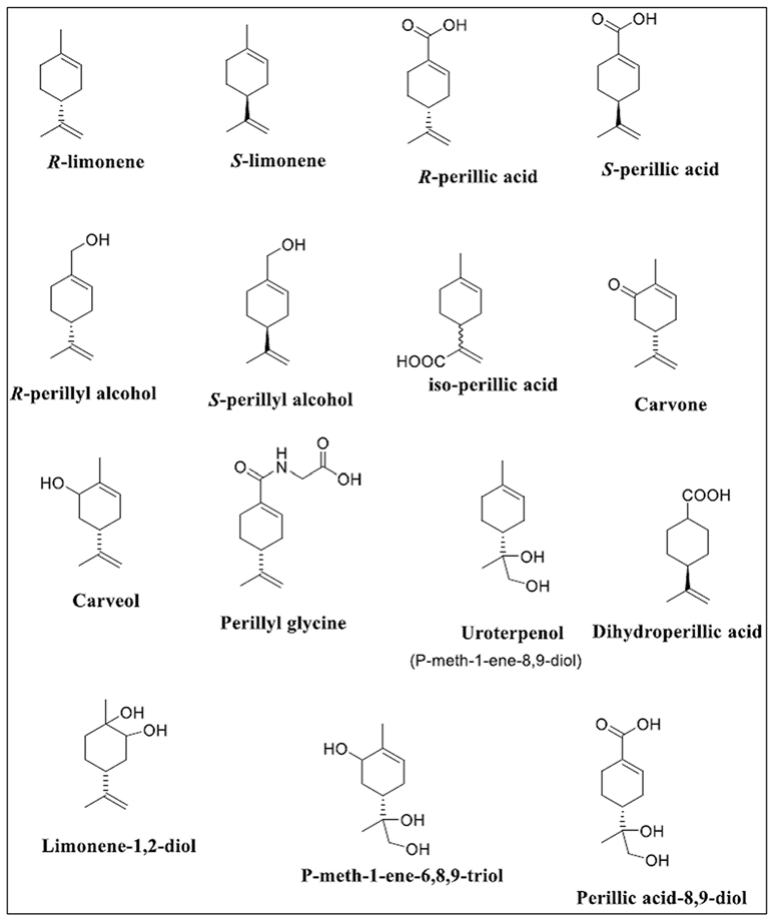
Structure of the monoterpene limonene and its metabolites

Emerging reports indicated that limonene might act as a prodrug because of the therapeutic potency of its metabolites, such as POH and PA have been found to be more effective agents [20,21]. This was evidenced in the determination of only the metabolites of terpenes in the plasma of chronically treated rats, but not the administered compounds, which suggested that the antitumor activity of terpenes may be mediated through their stable metabolites [22]. Although there are several metabolites of limonene (Figure 3), most of the current discussions are focused on POH. The interest in POH may be due to its initial evaluation in phase I and phase II clinical trials for the treatment of a range of cancers (breast cancer, ovarian cancer, and prostate cancer) [23–30] and its subsequent failure to show a clinical effect upon a phase II metastatic colon cancer trial conducted by the University of Wisconsin. Since the potency of POH is modest compared with many antitumor agents, its structural modifications has been carried out in recent times, and several kinds of POH derivatives synthesized with improved activities. Among these derivatives are POH carbamates [31], POH esters [32,33], POH glucosides [34,35], and amino-modified POHs [36].

As stated previously, it is well documented that limonene and its metabolites possess essential pharmacological bioactivities. However, these beneficial health potentials could be restricted by their low metabolic and plasma stabilities, low bioavailability, and tissue distribution [21,22]. This review therefore proposes that these features could be improved via effective drug delivery systems, prodrug approaches, and efficient nanoformulations. At present, there is existing research gap on pharmaceutical formulations of these monoterpenes and their synthetic derivatives with regards to the efficient nanocarriers viz., nanomicelles, liposomal encapsulation, self-nanoemulsifying drug delivery systems (SNEDDS), niosome nanoparticles, and nanoemulsion formulations that may help to inhibit first pass metabolism and enhance the therapeutic efficacy of these monoterpenes *in vivo*. Also, detailed pharmacokinetic and pharmacodynamic profiles of the newly synthesized derivatives have not been reported. Such studies could help to unearth the therapeutic efficacies of the active constituents for future clinical evaluations Moreover, structural modifications of limonene and especially its metabolites are promising in overcoming some of the obstacles hindering the bioactivities of these monoterpenes.

Inhibition of protein prenylation is currently recognized as the main molecular target associated with the anticancer activity of limonene and its metabolites [21,22]. However, most of the current synthetic derivatives of these monoterpenes [35,36] have not yet been evaluated for their inhibitory role against isoprenylation enzymes and other related molecular targets. Thus, further studies in this area of interest could result in the understanding of the molecular mechanisms associated with the antitumor activities of the derivatives while aiding in the discovering of novel relevant molecular targets necessary for developments of these monoterpenoids into effective anticancer drugs.

In the next sections, the biochemical significance of limonene and its metabolites is discussed with emphasis on their anticancer activities and the inhibitory effects against isoprenylation enzymes. Furthermore, this review also highlighted future prospects of these monoterpenes and their derivatives in drug design and development.

## Antitumor activities of limonene, perillyl alcohol, and perillic acid

In recent times, POH, limonene, and other dietary monoterpenes have demonstrated some degree of chemotherapeutic activity against lung, pancreatic, mammary, liver, colon, and prostatic tumor models [37–40]. It is worth noting that these monoterpenes are effective, nontoxic dietary antitumor agents which act through a variety of mechanisms of action and hold promise as a novel class of antitumor drugs for human cancer. Upon the administration of limonene and its metabolites, the growths of the aforementioned tumors were inhibited mainly through the induction of cytostasis and/or apoptosis [13,40,41]. The outcome of such inhibitions may alter signal transduction followed by changes in gene expression. Usually, the antitumor activity of the monoterpenes correlated with the differential expression of the growth and apoptotic genes necessary for tumor proliferation [38,39].

Apoptosis is known to be involved in a variety of biological events. Accumulating evidence suggests that most anticancer agents can trigger apoptosis in tumor cells *in vivo* and *in vitro*, which might be due to their effectiveness in prevention of tumor growth [42]. It is well known that caspases play the central role in apoptosis. Caspase-8 and caspase-9 are the initiator caspases with caspase-8 usually involve in the extrinsic death receptor apoptosis pathway, while caspase-9 has been linked to the intrinsic mitochondrial death pathway [43]. Both proteins tend to cleave and activate the downstream effector caspases, such as caspase-3, which cause poly(ADP-ribose) [PAR] cleavage and eventually lead to apoptosis.

It has been demonstrated that limonene, POH, and PA inhibited the proliferation of lung cancer cells (H322 and H838) with an increase in caspase-3 activity and cleavage of PAR [44]. In the same study, POH and PA were observed to elicit dose-dependent cytotoxicity, while inducing cell cycle arrest plus apoptosis with increasing expression of Bax, p21, and caspase-3 activity in both H322 and H83 cell lines [45]. Moreover, anticolon cancer effect of (*R*)-limonene was induced via apoptosis and modulation of polyamine metabolism [13]. Furthermore, Jia et al. [13] posited that increase Bax/Bcl2 ratio coupled with up-regulation of cleaved caspase-3, caspase-9, PAR, and cytochrome *c* demonstrated that limonene could induce mitochondrial dependent apoptosis in LS174T colon cancer cells.

The phosphatidylinositol 3-kinase/protein kinase B (PI3K/AKT) is an important intracellular signaling pathway, which plays a critical role in controlling survival and apoptosis. In many types of cancer this pathway is overactive, supporting cell survival and proliferation [45–47]. Several reports have shown that some anticancer agents induce apoptosis, in part, by blocking this pathway [48,49]. Activated Akt phosphorylates and inactivates several pro-apoptotic proteins, including Bcl-2-associated death promoter (BAD) [50], and caspase-9 [51], inhibiting the intrinsic apoptotic pathway. Recently, geraniol, an acyclic dietary monoterpene, was reported to induce apoptosis by inhibition of Akt signaling [52]. Jia et al. demonstrated that limonene decreased not only phosphorylated Akt protein levels but also Akt activity. Moreover, the authors observed that caspase-9, a downstream target of Akt, was cleaved to the active form by the limonene treatment. Collectively, these results suggested that inhibition of the Akt pathway contributed, at least in part, to the apoptotic cell death caused by the limonene treatment [13].

Nowadays, other studies have revealed that inflammation may be a key driver of cancer. Inflammation is an important event that is self-limiting and protects humans in times of infections and diseases. However, chronic inflammation has been associated with tumor progression through a network of proinflammatory mediators involved in complex signaling that aids tumor cells to utilize the circulatory system for distant tissues invasion and formation of cancers [53]. Schulz and colleagues revealed that POH alongside PA interfered with RAS/mitogen activated kinase (MAPK)-dependent interleukin-2 (IL-2) production in mitogen-stimulated T-cells and substantially suppressed IL-2 and IL-10 production in mitogen-activated T-lymphocytes. Also, they found that transforming growth factor-β1 and IL-6 generations were constant upon PA along with POH treatments, suggesting selectivity of these agents towards cytokine secretion. Further, H9 T-lymphoma cells exposure to PA resulted in a dose-dependent depletion of membrane-bound Ras proteins [54].

Aside the chemoprotective effect, (*S*)-PA also inhibited rat smooth muscle cells (SMC) proliferation as well as thymidine incorporation in a dose-dependent manner [55]. Additionally, (*S*)-PA inhibited DNA synthesis within a single cell cycle in simulated myocytes when added 8 h after the mitogenic stimulus [55]. While these observations are of interest, a better understanding of the underlying mechanisms of action of these monoterpenes is necessary toward future investigations into the potential biological/anticancer effect of these monoterpenes. Therefore, an effective design and synthesis of compounds bearing moieties of these agents could be explored. Of note, inhibition of protein isoprenylation has been the main molecular targets of through which these monoterpenes carried out their anticancer activities [56].

## Protein prenylation

Protein prenylation, the attachment of farnesyl (C15) or geranyl (C20) isoprenoid to one or more cysteine residues located near the C-terminus, are post-translational modifications that modulate protein cellular localization, signaling, and degradation [57]. Prenylation allows proteins to localize to the cell membrane where they can exert their function and interact with downstream effectors. Many of the key members of the Ras superfamily of proteins, including Rho and Rab guanosine triphosphatases (GTPases), required prenylation for correct functions. Three independent prenylating enzymes namely protein farnesyl transferase (FTase) and two protein geranylgeranyl transferases (GGTase1 and 2) are responsible for addition of respective isoprenoids. The subtle change in amino acid recognition sequence by these three enzymes ensures that any given protein is prenylated with only one of them. GGTase1 transfers geranylgeranyl diphosphate (GGPP) to proteins containing CAAL domain where C is cysteine, A can be any aliphatic amino acid, and L is always leucine. In contrast, GGTase2 recognizes proteins with C–C or CLC domain and prenylate them. To date, Rab family of proteins was the only known candidate to possess C–C or CLC domain [17,58].

Rho family of GTPases (about eight members) belongs to Ras superfamily of protein that is geranylgeranylated by GGTase1. Members of Rho family especially RhoA and Rac1 play a vital role in Ras mediated transformation of NIH 3T3 cells [59]. Furthermore, Ras prenylation, particularly farnesylation was targeted to prevent transformation of cells [60,61]. Farnesylation was effectively stopped by inhibiting FTase through peptidomimetic compounds. Though proven to be very good drugs with no side effects, FTase inhibitors (FTI) could not completely prevent tumor proliferation as some Ras isoforms like *K*-Ras-4B are resistant to FTIs like L-744,832, and FTI-277 or undergo alternative prenylation, i.e. geranylgeranylation [62]. On the other hand, most of the identified GGTase inhibitors have shown consistent results by arresting cells in G0/G1 phase and induce apoptosis [63].

It is well documented that several cancers are associated with the dysfunction Ras signaling. Nonetheless, prenyltransferase inhibitors have received much attention as potential anticancer agents. Limonene, POH, and their metabolites have been documented to inhibit the protein prenylating enzymes, protein farnesyl transferase and geranylgeranyl transferase (Table 1) [20,21,55,64].

**Table 1:** IC_50_ values for the inhibition of the isoprenylation enzyme activities by limonene, its metabolites, and standard compounds [20]

Compounds	FTase (mM)	GGTase (mM)
*R*-Limonene	>40	>40
*S*-Limonene	>40	>40
*R*-Perillic acid	8.1 ± 1.0	3.4 ± 0.3
*S*-Perillic acid	10.7 ± 0.9	4.1 ± 0.5
*p*-Menth-1,8-dien-10-oic acid	5.0 ± 0.8	2.6 ± 0.3
*R*-Carvone	1.5 ± 0.4	2.3 ± 0.5
*S*-Carvone	1.4 ± 0.2	7.0 ± 2.0
*R*-Perillyl alcohol	10.4 ± 1.5	2.1 ± 0.4
*S*-Perillyl alcohol	10.2 ± 2.0	1.9 ± 0.5
	**Positive controls (µM)**	
L 744832	0.1 ± 0.004	25 ± 8
α-Hydroxyfarnseylphosphonic acid	2.6 ± 0.24	25 ± 5
Chaetomellic acid	2.5 ± 0.5	40.0 ± 7

Each IC_50_ value is the mean of three independent experiments ± SD [20].

The results obtained in Table 1 depicted that limonene and its metabolites showed inhibitory activities against the isoprenylation enzymes, FTase, and GGTase in the cytosol of the rat brain. It can also be inferred from Table 1 that the two isomers of limonene (*S* and *R*) were only weak inhibitors. However, the major metabolites in the plasma of the rats, thus PA, *p*-Menth-1,8-dien-10-oic acid, and POH demonstrated remarkable inhibitory activity, with IC_50_ values in low mM. Moreover, the metabolites were stronger inhibitors of the GGTase1 enzyme than fernesyltransferase [20].

In addition, the results obtained in Table 1 indicated that limonene metabolites exhibited greater GGTase inhibition compare to the standard peptidic inhibitor L 744832. The preference of these monoterpenes for inhibition against GGTase support the evidence that POH anticancer effect stems from its ability to cause G0/G1 cancer cell cycle arrest via the putative inhibition of post-translational modification of signal transduction proteins involved in the Ras/MAPK pathway by depleting farnesylated Ras levels, an effect which may contributes to inhibition of IL-2 production in T-cell activation [54]. Therefore, limonene and its metabolites could be considered as novel class of chemotherapeutic/immunosuppressive agents, which stimulate a depletion of farnesylated Ras levels by a mechanism distinct from 3-hydroxy-3-methyl-glutaryl-CoA (HMG-CoA) reductase or FTase inhibition.

In another study, an oxygenated metabolite common to both the anticancer monoterpenes limonene and POH, PA methyl ester (PAME), was observed as the most potent inhibitor of yeast FTase and GGTase *in vitro* compared with the parent drugs [21]. Meanwhile, the assessment of the inhibitory role of most of the current synthetic derivatives of POH against GGTase and FTase has not been studied and therefore evaluations of such therapeutic effects need further investigations.

Besides the blocking of isoprenylation of small G proteins, (*R*)-limonene has also been observed to have a wide range of other cellular effects, including the inhibition of coenzyme Q synthesis [65], induction of various growth factors and their receptors [66] and induction of Phase I and Phase II carcinogen-metabolizing enzymes (cyt P_450_) [67]. For example, in the initiation phase of mammary carcinogenesis, chemopreventive effects of (*R*)-limonene were potentially due to the induction of Phase II carcinogen-metabolizing enzymes, which neutralized the toxicity of chemical carcinogens, and in the post initiation phase, tumor suppressive activity of *R*-limonene might be induced by inhibiting the isoprenylation of cell growth-regulating proteins such as Ras and apoptosis induction. Thus, the tumor suppressive activity of (*R*)-limonene might be induced by inhibiting the isoprenylation of cell growth-regulating proteins such as Ras and apoptosis induction [41].

## Impact of limonene and its metabolites on drug design and development: future directions

Several lines of evidence have confirmed that metabolites of limonene are the most active compounds rather than native limonene [68]. With short-half life and unstable concentration of limonene and its metabolites *in vivo*, there is the need to explore ways of improving the metabolic alongside plasma stabilities of limonene and/or its metabolites. This could be achieved by efficient drug delivery systems, prodrug approach and nanoformulations.

Cytotoxicity and other related side effects are the most serious problems associated with the currently available anticancer drugs [17]. Other limitations include widespread systemic distribution and rapid elimination of the administered anticancer drugs from the host body. Before agents like limonene, POH, and related compounds as could be accepted effective anticancer agents in the clinical setting, it is desirable to address some of the issues associated with their solubility, palatability, and sustained/controlled release in systemic circulation. This requires designing of suitable drug-delivery systems that can release the drug gradually over a long period of time and, in turn, facilitate its uptake by cancer cells and thereby helps in increasing the efficacy of the entrapped drug.

Recently, several natural bioactive compounds have been nanoformulated by our team and this has potentiated their antitumor properties [69–74]. Notably, Yi et al. improved the antitumor potential, oral bioavailability and tissue distribution of sterols separated from *Flamulina velutipes* via sterol-loaded microemulsion formulation. Interestingly, *in vitro* cytotoxic assay showed that after 72 h of treatments with the formulated drug demonstrating strong inhibitory effect against U251 cells, and was more efficacious than the standard anticancer drug, 5-fluorouracil (5-FU) [69]. The authors also formulated these sterols via liposomal encapsulation, and mixed micellar nanoformulation in which both formulations displayed enhanced antitumor, bioavailability, and biodistribution compared with the free sterols. These studies indicated the effectiveness of nanoformulations in potentiating the pharmacological effects of lipophilic drugs like limonene and its metabolites. Intriguingly, limonene, POH, PA and most of the current derivatives are water-insoluble and formulation could help improve their solubility, oral absorption, and their concomitant bioavailabilities. In a typical study, the bioavailability and the tissue distribution of perillylaldehyde (PAH), a derivative of limonene was enhanced through PAH-loaded self-nanoemulsifying (PAH-SNEDS) [75], and PAH-loaded liposomal nanoformulation (PAH-LNF) [76], which potentiated the antioxidant and antidiabetic activity of PAH than the free PAH [76].

Generally, entrapment of these monoterpenes in the aforesaid nanocarriers would offer the potential to enhance their therapeutic index, either by increasing the drug concentration in tumor cells and/or by decreasing the exposure in normal tissues [77,78]. Normally, a carrier plays two roles in cancer targeting therapy. One is to reduce the toxicity of the drug for the normal cells, and the other is to enhance the specificity of the drug to the target cells. After the prodrug enters the target cells, the drug is released from its inactive form (or less toxic form) to an active form and performs its physiological functions. For example, POH-bearing poly-lactic glycolic acid (PLGA) microparticles when administered to tumor-bearing animals caused greater tumor regression and increased survival rate (∼80%) as compared with the group receiving free form of POH (survival rate 40%) [79].

Research on derivatives of natural products has revealed new possibilities for therapeutic anticancer agents. More than two thirds of the drugs currently used in cancer treatments are derived directly from natural products, or have been developed using knowledge gained from the activities of their ingredients [80–82]. In an attempt to develop more potent drugs, many studies with natural products and their analogues have been conducted, showing antitumor properties of various plants and their constituents [83,84]. Monoterpenoid compounds with p-menthane structures are abundantly found in nature [85]. Several of these compounds, such as carvacrol [74], thymol [86,87], (*R*)-limonene [88], PA [89,90], PAH [91], and POH [92,93], have been studied for their anticancer potential. Others have also been reported to possess *in vitro* cytotoxic effects on cancer cell lines [83].

**Figure 4:**
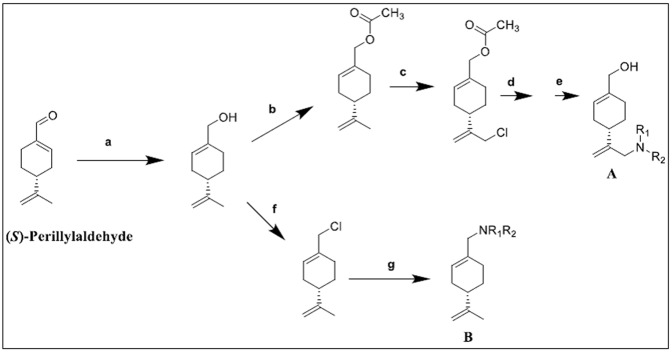
The synthetic routes, reagents, and conditions of the two series of amino-modified (*S*)-*POH* (**A** and **B**) (**a**) Sodium borohydride (NaBH_4_), ethanol (EtOH), 0°C, R.T., 3 h; (**b**) acetic anhydride (Ac_2_O), pyridine, r.t., 4 h; (c) sodium hypochlorite (NaClO), acetic acid (AcOH), 0°C, 0.5 h; (**d**) R1R2NH, potassium (K_2_CO_3_), EtOH, reflux, 8–12 h; (**e**) sodium hydroxide (NaOH), water (H_2_O), reflux, 2 h; (**f**) triphenylphosphine (Ph_3_P), tetrachloromethane (CCl_4_), dichloromethane (CH_2_Cl_2_), r.t.; (g) R1R2NH, K_2_CO_3_, acetonitrile (CH3CN), reflux, 6–8 h. R1R2NH denotes heterocyclic amine or an aromatic amine [36].

POH is considered as the most promising member of the group *p*-menthanes. As a compound with cytotoxic activity against a variety of cancer cells, it is believed that its structural modification could further enhance POH as an effective [16,29,94,95]. Chemical modifications of POH by medicinal chemists have recently seen several analogues of POH with improved anticancer activities. The compound perillaldehyde 8,9-epoxide (PAHE) is a synthetic derivative of structurally correlated POH. In a study of the percentage growth inhibition of cells (GI %) by Andrade and colleagues [96], POH and its derivative PAHE were evaluated against tumor cell lines of ovarian adenocarcinoma, colon carcinoma, and glioblastoma with PAHE demonstrating a higher *in vivo* GI%. The authors reported that the presence of epoxide and aldehyde functional groups may be a determinant for high cytotoxicity. Also, dehydroperillic acid, a derivative of POH obtained by biotransformation of POH with *Fusarium culmorum* exhibited improved anticancer activity by the induction of apoptosis in lung adenocarcinoma. Moreover, Nandurkar and co-experimenters synthesized POH-neoglycosides which showed improved anticancer effect on prostate cancer PC-3 and non-small cell lung cancer cells, A549 [35]. Furthermore, with the recognition that amino-modification has been proved to enhanced the solubility and concomitant anticancer activity of many natural products, such as that of comptothecin [97,98], β-eleminene [99], and limonene [100], Hui and co-workers designed and synthesized two series of amino-modified derivatives (**A** and **B**) of (*S*)-perillyl using (*S*)-perillaldehyde as the starting material (Figure 4) [36]. These derivatives showed increased antiproliferative activity in human lung cancer A549 cells, human melanoma A375-S2 cells, and human fibrosarcoma HT-1080 cells compared with that of (*S*)-POH [36].

The aforementioned findings suggest that structural modification could prospectively enhance the antitumor effect of limonene and its metabolites. Nonetheless, more studies are needed with regards to the pharmacokinetic profile and pharmacodynamics of the derivatives of POH that have demonstrated remarkable anticancer effect in order to facilitate their development for future clinical applications. Also, molecular docking could be explored in the identification of novel biological targets of these newly discovered derivatives of POH, which could aid in the designing and synthesis of more potent chemoprotective agents. Moreover, the drug-like properties of the synthesized derivatives of limonene and its metabolites are desired and could be used to predict their *in vivo* pharmacokinetics and pharmacodynamic properties of these compounds. Thus, medicinal chemists/drug designers may explore these monoterpenes as starting blocks to design novel bioactive compounds for the treatment of cancer, inflammatory, and other dysfunctions. Currently, literature has reported few anticancer mechanisms of action of limonene and its metabolites. Therefore, detailed research should be conducted on the mechanistic action of limonene, its metabolites and especially the derivatives of POH in order to identify pharmacological biomarkers involve in their chemotherapeutic effects.

For clinical development of these monoterpenes and their analogues, evaluation of the drug–terpene interaction against metabolic enzymes, especially cytochrome P_450_ subtypes is promising in establishing the clinical effects of these monoterpenes. Chen et al. [100] demonstrated that (*R*)-limonene and six other monoterpenes were substrates of cytochrome 2B6 with type I binding affinity. The authors revealed that the best substrate was α-terpinyl acetate, which inhibited the bupropion hydroxylation activity of P_450_ 2B6 [101]. Given their prevalence and their use in medicine, terpene–drug interactions could be a significant issue in clinics and therefore systematic studies is needed since terpenes play important roles in many foods, cosmetics, pharmaceutical, and biotechnological industries [102]. In this regard, the induction of cytochrome P_450_ enzymes by limonene, its metabolites and derivatives coupled with its effect on the anticancer activity of these monoterpenes is an area that needs further investigation.

## Conclusion

In summary, based on compelling evidences it can be concluded that limonene and its metabolites possess essential pharmacological bioactivities. However, these beneficial health properties could be limited by their low metabolic, and plasma stabilities, low bioavailabilities and tissue distribution/accumulation. This review therefore proposes that these features could be improved via effective drug delivery systems, prodrug approaches, and efficient nanoformulations. Moreover, detail mechanistic studies are also propounded as the possible means of unearthing pharmacological biomarkers for the chemopreventive and anti-inflammatory activity of limonene, its metabolites, and synthetic derivatives. Furthermore, the prospect of modifying limonene and its metabolites to serve as prominent scaffolds in designing novel potent bioactive compounds was discussed.

## Abbreviations

Bcl-2: B-cell lymphoma-2
FTase: farnesyl transferase
GGTase: geranylgeranyl transferase
IL-2: interleukin-2
PA: perillic acid
PAH: perillylaldehyde
PAHE: perillaldehyde 8,9-epoxide
PAR: poly(ADP-ribose)
POH: perillyl alcohol
TGF-β: transforming growth factor β
